# Decentralized IoT Data Authentication with Signature Aggregation

**DOI:** 10.3390/s24031037

**Published:** 2024-02-05

**Authors:** Jay Bojič Burgos, Matevž Pustišek

**Affiliations:** Faculty of Electrical Engineering, University of Ljubljana, 1000 Ljubljana, Slovenia; matevz.pustisek@fe.uni-lj.si

**Keywords:** blockchain, IoT, data authentication, signature aggregation, rollup, SNARK

## Abstract

The rapid expansion of the Internet of Things (IoT) has introduced significant challenges in data authentication, necessitating a balance between scalability and security. Traditional approaches often rely on third parties, while blockchain-based solutions face computational and storage bottlenecks. Our novel framework employs edge aggregating servers and Ethereum Layer 2 rollups, offering a scalable and secure IoT data authentication solution that reduces the need for continuous, direct interaction between IoT devices and the blockchain. We utilize and compare the Nova and Risc0 proving systems for authenticating batches of IoT data by verifying signatures, ensuring data integrity and privacy. Notably, the Nova prover significantly outperforms Risc0 in proving and verification times; for instance, with 10 signatures, Nova takes 3.62 s compared to Risc0’s 369 s, with this performance gap widening as the number of signatures in a batch increases. Our framework further enhances data verifiability and trust by recording essential information on L2 rollups, creating an immutable and transparent record of authentication. The use of Layer 2 rollups atop a permissionless blockchain like Ethereum effectively reduces on-chain storage costs by approximately 48 to 57 times compared to direct Ethereum use, addressing cost bottlenecks efficiently.

## 1. Introduction

In the Internet of Things (IoT) realm, ensuring device identity and data authenticity is highly important. Data authenticity refers to the veracity and non-repudiation of data, ensuring that they have not been tampered with or forged. Data authentication and integrity are important for several reasons. Firstly, they safeguard the reliability and trustworthiness of data, ensuring that they accurately reflects the real-world conditions they represents. This is particularly critical in Industrial Internet of Things (IIoT) scenarios [1], where data are used to make informed decisions about critical assets, such as manufacturing equipment or power grids. Invalid or manipulated data can lead to erroneous actions, potentially causing disruptions, safety hazards, or financial losses.

The growing trend of data sharing and monetization within the IoT landscape underscores the need for robust data authenticity mechanisms. This is because companies and individuals increasingly exchange or purchase IoT data for various applications, such as predictive maintenance, smart home optimization, or healthcare analytics [2,3,4]. Data authenticity also plays a crucial role in supporting the development of artificial intelligence (AI) applications [5]. AI algorithms rely on vast amounts of high-quality data to train and refine their models. If the underlying data are not authentic, the AI models may generate erroneous or misleading insights, potentially leading to flawed decisions and actions.

Blockchain technology, a distributed ledger system known for its transparency, security, and immutability, offers a promising solution to address the challenges of IoT data authentication. By leveraging blockchains’ inherent features, organizations can establish a trustless environment where IoT data can be securely shared and verified without intermediaries [6].

Blockchain-based IoT data authentication solutions can enable several valuable features, including the following:

- Data Integrity: Blockchains’ tamper-proof nature ensures that IoT data remain unaltered throughout their lifecycle, preventing unauthorized modifications or data breaches.
- Device Authentication: A blockchain can serve as a secure repository for device identities, enabling reliable authentication of IoT devices and preventing unauthorized access or data injection from malicious or compromised devices.
- Provenance Tracking: Blockchains’ ability to track data origin and movement provides traceability for IoT data, allowing for the verification of data sources and provenance.

However, while blockchains offer significant benefits for IoT data authentication, they also presents challenges that must be addressed. Scalability and cost are two significant issues that must be carefully considered [6]. The resource-constrained nature of many IoT devices raises concerns about the computational overhead and energy consumption associated with blockchain transactions. Moreover, the cost of maintaining and operating blockchain networks could be prohibitive for certain IoT applications.

The referenced paper [7] examines the application of permissioned blockchain architectures within a data authentication framework for IoT. Our stance is that relying solely on such architectures introduces limitations that could restrict the framework’s scalability and broader applicability. While efficient and controllable, these systems risk segregating IoT networks from the dynamic and technologically rich broader blockchain ecosystem, which boasts considerable liquidity and a substantial user base. Additionally, private blockchains necessitate the establishment of validators, adding layers of complexity and an element of centralization, which runs counter to the ideal of a decentralized system. A fully blockchain-based data authentication solution for IoT devices based on public blockchains might also be impractical due to the prohibitive costs involved.

To address these challenges, our proposed framework takes a different approach from conventional blockchain-based authentication methods, which typically require direct interactions between resource-limited IoT devices and blockchain nodes [8]. We propose a layered architecture that decouples the generation and authentication of IoT data from the blockchain itself. In this model, IoT devices transmit their data to edge aggregating servers. These aggregators, acting as intermediary nodes and are equipped with ample computational resources to verify IoT signatures. Only the aggregated signatures/proofs or their hashes are relayed to the blockchain, serving as timestamps for the IoT signature-verification process.

This approach offloads the computational burden from IoT devices and creates a highly modular and scalable system. On the blockchain side, we employ scalable L2 blockchain scaling solutions, such as rollups, to address the issue of blockchain transaction throughput. By leveraging these scaling solutions, the blockchain can handle a significantly higher volume of IoT data without compromising security or performance. To further enhance scalability, edge aggregating servers, which collect and aggregate signatures from multiple IoT devices, are introduced. The increase in IoT devices and the scalability issues that these present can now be solved by introducing more aggregators and/or new rollups on the blockchain side.

- Our framework prioritizes data privacy by keeping sensitive IoT data confidential. Instead of allowing IoT devices to communicate directly with the blockchain, we handle all data verification through edge aggregator servers. This approach leverages Zero-Knowledge Proofs (ZKPs), enabling us to verify data without exposing the actual information, thus enhancing data privacy even further.

The implemented framework enabled us to conduct realistic experiments, providing detailed insights into the operation of a trusted, decentralized IoT solution. This solution stands out for its emphasis on data authenticity and the incorporation of Zero-Knowledge privacy principles. The key research questions investigated in this paper are as follows:

- Impact of different proving systems: Our exploration delves into various proving systems, examining their impact on the efficiency of proving and verification times and the sizes of the proofs required to ensure efficient data authenticity. Within our solution, we observed a shift in the performance bottleneck. It moves from the blockchain network to an off-chain system, which is particularly evident during the proving and verification stages.
- On-chain storage costs: The research also includes an assessment of the financial implications of using public blockchain networks for data storage. This component is vital to maintain a high level of decentralization and trust, providing a clear advantage over private or consortium blockchain networks. Our analysis balances the need for decentralization with the practical aspects of storage costs on public blockchains.

The contributions of our work can be summarized as follows:

- Development of a novel framework: We introduced a unique framework that integrates edge aggregating servers with Ethereum Layer 2 rollups. This design enhances scalability and security in IoT data authentication, minimizing the need for continuous direct interactions between IoT devices and the blockchain. By recording essential information on L2 rollups, our framework ensures data verifiability and trust, creating an immutable and transparent authentication record.
- Analysis of the viability of proving systems for our framework: Our work explored and compared the Nova and Risc0 proving systems, focusing on their efficiency in proof generation and verification times, as well as the proof sizes required for data authenticity. We discovered that employing proof recursion and compression is crucial for achieving superior performance in authentication, surpassing the efficiency of proving a single signature verification, and even outperforming methods like ECDSA batch verification.
- Cost-effective on-chain storage solutions: We assessed the financial implications of using public blockchain networks for data storage. The use of Layer 2 rollups led to significant reductions in on-chain storage costs. Our findings reveal that leveraging rollups atop public blockchains like Ethereum offers low fees while keeping us integrated within the larger, open-source public ecosystem, preventing isolation from the broader market.

The paper begins with an Introduction highlighting the importance of data authenticity in IoT and the potential of blockchain technology to address related challenges. Section 2, the Literature Review, overviews existing IoT data authentication approaches and briefly presents key fundamentals of our research as layered blockchain architectures and proving systems. Section 3 details the proposed solution, dividing it into off-chain and on-chain components. Section 4, Results, presents the findings from our tests on proving times, verification times, proof sizes, and on-chain storage costs. Section 5 discusses these results. Section 6 presents a set of conclusions summarizing the key insights and indicates possible future research.

## 2. Literature Review

Since our goal is to establish a framework for IoT data authentication that does not depend on permissioned blockchain networks or centralized solutions—which often entail reliance on third parties and carry inherent trust limitations—this section provides an overview of existing approaches to IoT data authentication. We also present the concept of layered blockchain structures and explore proving systems. These elements are crucial for the implementation of our proposed solution.

### 2.1. Existing Approaches to IoT Data Authentication

Data authentication plays an important role in IoT systems with some key challenges, including the following:

- Device authentication (registration and identity management) : Essential for preventing rogue device infiltration and ensuring data originate from verified sources, thereby maintaining IoT ecosystem integrity [7].
- Data integrity: IoT data must remain unchanged during transmission and storage to ensure their reliability [8].
- Data privacy: Protecting the confidentiality of sensitive information collected with IoT devices [9].
- Cyber security: IoT systems are vulnerable to attacks like DDoS, Sybil, and eavesdropping, which can disrupt operations and compromise data integrity [10,11].

Building on the understanding of these challenges, we find that while numerous solutions have been proposed, few manage to address all aspects of IoT data authentication in a decentralized manner.

The paper [8] proposes a solution for ensuring data integrity stored in cloud environments, particularly for Internet of Things (IoT) applications. The key idea is to use cryptographic hashes and smart contracts within a blockchain framework, utilizing Ethereum as the underlying blockchain. When data are stored in the cloud, a unique cryptographic hash of the data are generated and recorded on the blockchain. This hash serves as a digital fingerprint. Smart contracts are then used to verify the integrity of the data automatically. When data are retrieved, their hash is recalculated and compared with the hash stored on the blockchain. If the hashes match, it confirms that the data have not been altered, ensuring their integrity. While the approach presents a simple yet effective way to achieve data integrity, there are limitations to this approach. Not only does the use of Ethereum present considerable scalability issues since the costs would be too high, but the system focuses solely on verifying whether the data have been altered while in storage. It does not provide insights into the authenticity of the data themself. For instance, it does not verify whether the data originated from the correct IoT device or if they were authentic at the point of creation. This means that while the system can assure that the data have not been tampered with since they were stored, it cannot guarantee their initial authenticity or source. Therefore, additional mechanisms would be needed to validate the data’s origin and authenticity before they are stored and hashed on the blockchain.

In contrast to this approach, another intriguing solution is presented in the paper [12], which discusses combining edge computing with a blockchain to enhance authentication in IoT networks. Edge servers are primarily responsible for authenticating IoT devices by verifying their credentials against blockchain-stored data, ensuring network access is limited to authorized devices only. Beyond authentication, these edge servers also contribute to efficient data management. Edge computing is crucial for real-time data processing in IoT applications, reducing latency and bandwidth demands. However, the system does place a certain level of trust in edge servers. While the blockchain ensures the integrity of the data it stores, the accuracy and security of the data initially provided by edge servers are vital. The system employs advanced cryptographic methods, including elliptic curve cryptography, to enhance security and protect against data compromise. Therefore, while blockchain technology ensures data immutability, the overall reliability and security of the system hinges on the trustworthiness and security measures implemented at the edge-server level.

A similar approach is the DIoTA framework [13], which offers a layered decentralized ledger architecture to enhance IoT data authenticity. It employs a unique edge–global structure, where each edge ledger serves specific IoT devices, and a global ledger interlinks these edge ledgers. This setup facilitates efficient cross-ledger data verification and incorporates a lightweight data authentication scheme to reduce the computational load on IoT devices. However, despite its innovative structure, DIoTA encounters significant limitations. Its reliance on a permissioned blockchain undermines the true essence of decentralization. The framework necessitates the establishment of validators, typically operated by entities within the system, such as IoT device owners or data analytic service providers. This model drifts away from a fully decentralized approach, as it centralizes control to a certain extent. If the number of these validators is limited or if they are heavily concentrated within a few entities, the system’s security and decentralization are compromised.

On the positive side, DIoTA introduces some valuable concepts. For instance, the use of efficient data authentication methods for IoT communication with the ledger, specifically Hash-based message authentication code (HMAC) or Cypher-based message authentication code (CMAC), are noteworthy. This aspect could be integrated into our approach to enhance the efficiency of IoT device interactions, maintaining data integrity while minimizing resource utilization.

ZeroTrustBlock [14] presents a similar solution in the healthcare field that utilizes a permissioned blockchain based on a Hyperledger to enhance the security, privacy, and interoperability of sensitive medical data with existing approaches, akin to IoT data authentication.

Another paper [15] explores an approach to integrating blockchain technology with IoT data authentication, specifically focusing on smart grids and smart meters. This method incorporates Zero-Knowledge Proof (ZKP) to enhance anonymity and secure data within the blockchain environment. The solution involves using blockchain technology and Zero-Knowledge Proof to secure data from smart meters within a smart grid environment. This approach aims to prevent data counterfeiting and personal information infringement. The system utilizes Ethereum’s smart contract functionality, incorporating Zero-Knowledge Proof to ensure data integrity and confidentiality. A significant drawback of this approach is that it requires IoT devices to write data directly onto the blockchain. This method is impractical and expensive due to the high financial costs of blockchain transactions.

While ZKP provides a method to verify the accuracy of information without revealing the data itself, the paper’s approach seems more focused on post-storage data integrity rather than authenticating incoming data from IoT devices. There is a lack of a clear link between continuous data generation by IoT devices and the initial two values used during registration, potentially allowing fabricated data to be sent to the server without a way to verify its authenticity against the initially registered values.

In the context of enhancing security in IoT environments, Xu et al.’s study introduces a significant development with their Certificateless Aggregate Signature (CLAS) scheme [16], which is meant to be used for smart home applications. This scheme, addressing security concerns and the issue of private key leakage of similar schemes, operates in a certificateless environment. It aims to simplify key management and reduce computational overhead, especially in scenarios with numerous interconnected devices and substantial data flow. However, the inherent possession of individual keys in many IoT applications raises questions about the necessity and practicality of transitioning to a new scheme like CLAS.

In contrast, our approach, leveraging the widely used ECDSA in conjunction with zk-SNARKs, offers a more direct ‘plug and play’ solution, obviating the need to adopt a different scheme. Our method, using ZKPs, also stands out in terms of privacy preservation. It is nonetheless an interesting approach, since the use of a different schemes like CLAS actually allows the aggregation of signatures, while our approach creates a proof of signature verification as the aggregate.

The solution in [17] describes a security method for Internet of Things (IoT) networks that focuses on making sure each device (I-Node) can be trusted before it sends information through the network. It does this by setting up a system where devices have to prove their identity using a digital signature. The network selects certain devices (I-G Nodes) to act as checkpoints, collecting and verifying information from other devices. These checkpoints then send all the verified information to a central point (data center) for storage.

However, this system introduces unnecessary complexity by imposing verification duties on I-G Nodes without clear incentives or benefits for their participation. A more streamlined approach might involve edge servers that manage the computational tasks, thereby reducing the burden on individual IoT devices/nodes and simplifying the network’s architecture. The proposed solution seems to occupy an awkward middle ground between decentralized and centralized systems, potentially inheriting the drawbacks of both without reaping the full benefits of either.

On the other hand, the work presented in [18] proposes a method to enhance privacy and computational efficiency in Industrial IoT systems. Utilizing the Paillier cryptosystem for privacy preservation and the Elliptic Curve Digital Signature Algorithm (ECDSA) with batch verification, the scheme addresses the critical need for secure data aggregation in edge-supported environments.

However, while this solution offers advancements in privacy protection, it also introduces additional computational work beyond ECDSA signing that may be prohibitive for IoT devices with limited resources. Furthermore, the use of batch ECDSA verification, as presented in [19], introduces a novel approach to authentication similar to ours but does not achieve the same performance. For instance, our system takes only 7.13 s to verify 100 signatures. In contrast, the batch verification in the cited work is limited to a maximum of 64 signatures and has verification times consistently exceeding 50 s when signatures originate from different devices. Although innovative, homomorphic encryption might not be necessary in scenarios where data are securely transmitted to trusted edge servers. Secure communication channels like SSL and TLS can provide adequate privacy without the extra computational overhead.

#### Discussion of Cited Studies and Their Implications

In our literature review of various IoT data authentication and privacy approaches, we identified several areas where these methods fall short, especially when compared to our approach. Key shortcomings in existing approaches include the following:

- Limited Post-Storage Verification: Some studies focus primarily on post-storage data integrity, overlooking the critical aspect of data-origin authentication.
- Over-Reliance on Edge Servers: Certain methods depend heavily on edge servers for data authentication without a clear mechanism to verify the correctness of the work carried out, raising potential security vulnerabilities.
- Permissioned Blockchain Limitations: Some frameworks utilize permissioned blockchains, which compromise the ideal of full decentralization. Additionally, when public blockchains are used, they often rely on expensive platforms without employing scaling solutions.
- Impractical Blockchain Interaction for IoT Devices: Other solutions require direct blockchain interactions using IoT devices, leading to high computational and financial costs.
- Practicality Concerns in Key Management: There are systems proposing new key management schemes that may not be practical for diverse IoT contexts. The challenge lies in adopting these new schemes as opposed to using widely used ones.
- Computational Overhead from Cryptosystems: Certain solutions, while enhancing privacy, add a significant computational workload, rendering them unsuitable for resource-constrained IoT devices.

### 2.2. Layered Blockchain Structure

The blockchain industry has witnessed a paradigm shift toward a layered or modular structure, notably in the public blockchain domain, such as the Ethereum ecosystem [20]. Traditional blockchain networks often employ a monolithic structure where a single chain processes and stores all transactions. While straightforward, this approach can lead to congestion and scalability issues, particularly when maintaining a high level of decentralization is a priority. In scenarios where decentralization is less critical, scalability can be enhanced by reducing the degree of decentralization. Nonetheless, as more users and transactions join the network and the emphasis on decentralization remains high, the system’s capacity to process transactions efficiently is strained, leading to higher fees and slower transaction times.

Private, permissioned, or consortium networks have emerged [21], offering privacy and customization. They also enable enhanced scalability by reducing the number required for consensus. However, this shift towards more centralized control can compromise large-scale decentralization’s security and resilience. Additionally, these networks often lack interoperability, isolating them from other blockchain ecosystems. This isolation results in a disconnect from public networks’ innovation, liquidity, and extensive user base. This is why public networks like Ethereum have evolved to adopt a layered blockchain architecture. This includes Layer 1 (L1) networks, providing security and decentralization, and Layer 2 (L2) solutions that offer customizability and scalability by processing transactions off the main chain (L1). This layered structure can scale further into Layer 3 scaling solutions by building upon L2 solutions like rollups. This layered approach facilitates a modular and interoperable ecosystem that benefits from broader blockchain space innovations and liquidity while catering to diverse user and application needs.

#### Layer 2 Scaling Solutions

Multiple L2 scaling solutions exist, with rollups being the most versatile and widely used [22]. Rollups achieve scalability and lower fees by processing transactions off L1 and then consolidating batches of these transactions into a single transaction that is recorded on L1. This shared cost mechanism significantly reduces individual transaction fees. Rollups can be somewhat centralized but still inherit robust security from L1, though decentralized configurations are also possible. Additionally, rollups can be modified for a particular use case, such as allowing private data to be stored on it, essentially creating an alternative to permissioned blockchains [23].

There are generally two types of rollups: optimistic rollups and Zero-Knowledge (ZK) or validity rollups. Their primary difference lies in the type of proof they utilize. Optimistic rollups operate on the assumption that all transactions are valid unless proven otherwise, offering a reward for identifying invalid transactions. This economic incentive model, however, requires a grace period for challenges, slightly delaying transaction finality. ZK rollups, on the other hand, use complex mathematical proofs (zk-proofs) to validate transactions. They offer quicker finality and more efficient data compression, albeit with current limitations in their ability to handle complex transactions [22,24,25].

Rollups are central to our solution, offering an easier setup than permissioned ledger systems. They do not necessarily require setting up individual validators, as L1 guarantees security via proofs. This reduces complexity and enhances scalability.

### 2.3. Proving Systems

Proving systems are fundamental in cryptography and blockchain technology. They are used to establish the validity of statements while potentially preserving the confidentiality of underlying data. In these systems, one party, known as the prover, convinces another party, the verifier, of the truthfulness of a given statement. There are two primary types of proofs to consider:

- Validity Proofs: In these proofs, the prover demonstrates the correctness of a statement or compliance with a specific condition [26,27].
- Zero-Knowledge Proofs (ZKPs): A specialized form of validity proof, ZKPs enable a party to prove the truth of a statement to another party without revealing any additional information beyond the fact that the statement is true. This characteristic is particularly important for preserving privacy in various applications [26].

While these two categories encompass a wide array of proof types, a particularly intriguing variant within this spectrum is SNARKs, standing for “Succinct Non-Interactive Arguments of Knowledge.” SNARKs are a distinctive type of cryptographic proof, marked by their two main properties: succinctness and non-interactivity. Succinctness means that the proofs are relatively compact and quick to verify, which is highly advantageous in numerous applications. Non-interactivity is a crucial feature where the prover can generate a proof without ongoing communication with the verifier.

Multiple trade-offs exist in various SNARK implementations, such as proof size, proving time and verifying time. Quantum resistance is also an important factor worth looking at [26,28].

Another important distinction is whether a SNARK is transparent or non-transparent. Non-transparent SNARKs require a process known as a trusted setup. This setup involves generating initial parameters (or keys) for proving and verifying systems. These parameters must be generated securely and trustworthy because any compromise in this process can lead to significant vulnerabilities. The trusted setup generates sensitive information which if not properly discarded, or worse, falls into malicious hands, can be used to create fraudulent proofs that appear valid. Therefore, the integrity and security of non-transparent SNARKs heavily rely on the proper execution and confidentiality of the trusted setup.

Transparent SNARKs, on the other hand, do not require a trusted setup. Instead, they use cryptographic techniques that avoid the need for a preliminary phase where sensitive parameters are generated. They are generally considered more robust and trust minimized, as they do not rely on the secrecy and integrity of a setup ceremony.

#### 2.3.1. Snark Composition, Aggregation, and Recursion

Different proving systems offer distinct advantages and trade-offs, such as speed in generating proofs, compactness of proofs, or efficiency in verification times. SNARK composition is a powerful technique that combines these strengths to optimize all aspects of proving and verifying. In SNARK composition, multiple SNARKs are strategically merged to address their individual limitations and enhance their strengths. For example, an inner circuit with a larger size might be used to prove a computation quickly, though it might result in a larger proof size. This larger proof then becomes the input for an outer, smaller circuit designed to verify the validity of the initial statement or computation. Although the proving time for this system might be slower, its smaller circuit size and reduced proof size offer a balanced solution with relatively fast overall proving and a small final proof.

This method also enables self-composition, where a SNARK’s output is recursively applied to itself. This iterative process gradually reduces verification overhead, making it particularly valuable in scenarios where verification demands more resources than proof generation [26]. SNARK composition enables significant applications like Incremental Computations and Proof Aggregation. Incremental Computations use SNARKs to verify the correctness of each step in a longer computational process, ensuring integrity throughout. Proof Aggregation involves combining multiple proofs into a single, more manageable proof, which is especially useful in scenarios requiring collective verification of numerous individual proofs.

**Figure 1:**
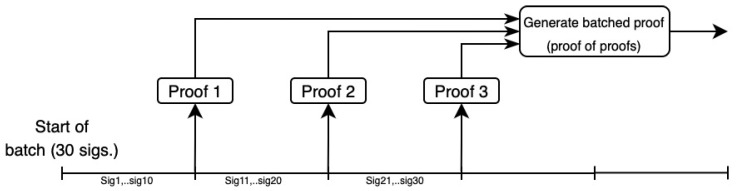
Proof aggregation.

Figure 1 illustrates how proof aggregation might be applied to batch signature verification. The naive approach would be to accumulate a large number of signatures before verifying them and create a single comprehensive proof of verification. With proof aggregation, proofs can be generated more promptly, and all the created proofs would then be aggregated, essentially creating a “proof of proofs”.

**Figure 2:**
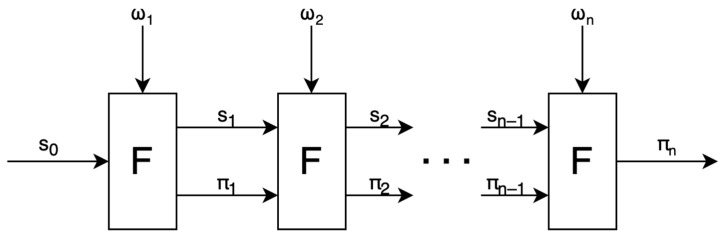
Incremental verifiable computation.

Recursive proofs, on the other hand, involve iteratively applying a function F (such as signature verification) to a computation with private inputs, where each step given private inputs (ω) generates a proof (π) confirming the accuracy of the current computation relative to the previous state (s_n−1_). Known as Incrementally Verifiable Computation (IVC), this method reduces memory usage by generating smaller, individual proofs at each step instead of a single, extensive proof or aggregating multiple proofs into one (Figure 2).

However, this approach also presents challenges. Since each new proof must validate the correctness of the previous proof, the process becomes computationally intensive. Despite the approach’s power and flexibility, creating and verifying a proof at every step increases the overall computational burden.

The Nova prover [29,30] introduces an innovative solution to the challenges of recursive proofs. Nova’s technique involves composing proofs throughout the computation but only finalizing them at the end. This strategy substantially reduces computational overhead, as it incurs the cost of proof generation just once.

Nova employs a process called Statement Folding to merge multiple instances into a single instance for verification. In this method, the prover P constructs a proof for a circuit C without needing to run the entire verification algorithm V(Vk, X, π), which typically involves the verifier key Vk (a part of the public parameters), the statement X, and the proof π, with the output being true or false. Nova simplifies circuit C by removing most verification checks, meaning that the prover only needs to construct a proof for a few selected checks, greatly simplifying the proof construction process. While Nova benefits from needing to verify only the last compressed SNARK, it also must ensure the correct execution of the folding process. This verification is integrated into the computed and proved function, typically referred to as the accumulator, which essentially accumulates and verifies that the folding was indeed completed correctly.

#### 2.3.2. Signature Aggregation

Since our solution focuses on removing interactions between IoT devices and the blockchain, there is a need for an alternative approach to signature verification. Traditionally, this was conducted by verifying the IoT signature directly on-chain and storing the actual signed data/messages off-chain. This approach was employed to lower data storage costs on-chain, but signatures were not eliminated. They still needed to be recorded on-chain and verified via a smart contract.

Signature aggregation allows us to remove even the signature verification from the chain. Taking care of the signature verification and data storage off-chain drastically lowers costs and paves a new way for optimization that does not require as much intervention with blockchains. It essentially moves the bottleneck from the blockchain to an off-chain system that can be further scaled using traditional means, even if that means adding extra hardware. This flexibility is not possible on current blockchains, where we are limited by the performance of any single chain.

In essence, signature aggregation is a method where multiple signatures from different sources (in this case, IoT devices) are combined into a single, compact signature. This process significantly reduces the amount of data required to verify the authenticity and integrity of messages from multiple devices. A common approach in blockchains, for example in Ethereum, is BLS signature aggregation [31], which allows the reduced signature size to be verified against the aggregated public key, created by combining all the public keys from the IoT devices.

While this approach is interesting, the fact that the majority of IoT devices use elliptic curve cryptography (ECC), specifically ECDSA, necessitates a different approach to aggregation. This is where SNARK aggregation comes into play, allowing us to replace many signatures with a single SNARK proof that validates the authenticity of all signatures.

As mentioned in Section 2.3.1, the Nova prover demonstrated how we could recursively prove any computation. In this case, the function F we are recursing would be the signature-verification algorithm. However, proving any computation is not trivial, and it is computationally more intensive than running the algorithm on its own. Modifying the signature verification to be more efficient, therefore, provides considerable performance gains. The efficient ECDSA verification method from Personae Labs [32] achieves just this. The performance benefits stem from executing certain computations outside of the proving phase. In essence, it is an optimized process that restructures the ECDSA signature verification so that parts of it are computed off the SNARK circuit. This method works by rewriting the ECDSA signature-verification equation to isolate elements that can be computed outside the SNARK, thus reducing the number of operations within the SNARK itself.

## 3. Framework Design

Our proposed framework enables a performant, secure, and privacy-focused IoT data authentication and storage method, utilizing permissionless blockchains and Zero-Knowledge proving systems. This framework achieves its goals by implementing a secure data validation process that minimizes the computational burden on IoT devices, distinguishing our solution with its performance-oriented design and adaptable architecture. It is set up to scale effectively, capable of accommodating an increasing number of IoT devices and data volumes. This scalability is achieved either by adding new L2 rollups or expanding the network of edge aggregating servers, ensuring the system adapts and grows without compromising performance, security, or cost-efficiency.

The solution comprises two main components:

- Off-chain components include IoT devices and edge aggregating servers. The aggregators serve as the first point of data aggregation and authentication, collecting and validating data from IoT devices, thus preparing it for on-chain integration. This process significantly reduces the computational load on individual IoT devices and decreases the need for direct blockchain interaction.
- On-chain components feature a layered blockchain structure and smart contracts built atop each L2 network. Ethereum is the foundational Layer 1 (L1), ensuring secure and reliable operations, while Layer 2 networks, such as Optimism Rollups, provide scalability and efficiency. Smart contracts facilitate IoT and edge-server registration and data storage.

### 3.1. Off-Chain

In this subsection we will delve into the off-chain part of our solution in more detail. Starting with IoT devices and data authentication via signatures and later using a proving system on the edge servers to aggregate signatures. In terms of IoT devices, we assume that the devices themselves are secure from a hardware perspective and/or on the software level, allowing the safe storage and use of private keys and data generation. Similarly, for edge aggregating servers, we also assume security at both the hardware and software levels, which is crucial for the effective operation of the proving system and the reliable aggregation of signatures.

#### 3.1.1. IoT Devices

To enable data authentication, all IoT devices need to do the following two tasks:

- Initial on-chain registration (only once);
- Data authentication via digital signatures.

Blockchains offer a solution that prevents data manipulation because of the immutable nature of blockchains. This is why every IoT device has to authenticate itself on the ledger, resulting in the creation and storage of its certificate, which includes its public key and other possible identifying details (e.g., what type of IoT device it is and what data does it produce). These certificates (public keys) are used to make sure that the IoT devices indeed generated the data.

Some IoT devices might be resource constrained, so expecting all IoT devices to communicate directly with a chain for anything else besides initial registration is not viable, since it comes with additional financial costs (paying for transaction fees).

To alleviate IoTs from this job, we introduce edge servers that aggregate IoT signatures into one single signature/proof. The signatures are thus essential for our solution, where we expect IoT devices to sign their messages before sending them to the edge aggregating server. Using digital signatures on the IoT side with verification on the edge aggregator offers faster data authentication. However, it increases the computational load on the IoT device, which may not be ideal for resource-constrained devices, such as those running on battery power. For these resource-constrained devices, Hash-based message authentication code (HMAC) is available, which reduces the computational burden on IoT devices by sacrificing the speed of data authentication.

#### 3.1.2. Edge Aggregating Servers

Our solution employs cryptographic proofs (SNARKs) and the algorithm used for ECDSA signature verification within the Nova prover. This enables a coprocessor of sorts on top of the blockchain. These coprocessors, or edge aggregators, allow for the offloading of computation and storage burdens from the blockchain to off-chain systems. This approach helps to decouple unnecessary functions from the ledger, moving bottlenecks to off-chain systems where they are more manageable. The ECDSA verification algorithm checks the validity of signatures, and the Nova prover confirms that the algorithm has been executed correctly and on valid inputs, including signatures, messages, and public keys. The resulting proof serves as an aggregate verification of the signatures’ authenticity. It is important to note that while this method increases overheads on off-chain systems, it is more feasible than handling these tasks on the blockchain, where resources are more limited.

The operational steps each edge aggregator has to make are as follows:

- Initial on-chain registration (only once).
- Signature pre-processing (conform with efficient ECDSA).
- Building a Merkle tree for each batch (includes public keys, signatures, messages, and batch identifiers) ensures all relevant data are accounted for in a verifiable structure.
- The edge aggregator signs the Merkle root along with the batch number and uses this as the public input to the prover. This ties the proof generation directly to the specific dataset represented by the Merkle tree.
- Writing each batch’s Merkle root, proof hash, and batch identifier on-chain. It enhances transparency and provides an immutable record that can be independently verified.

##### Aggregating Signatures

All edge aggregators must register on-chain the same way IoT devices do. This is because they are responsible for aggregating the signatures and writing on-chain, so we need a way to verify that the right aggregator responsible for a set of IoT devices has indeed completed the work and published the relevant data (for authentication) on-chain.

At the core of our system is the process of data collection from IoT devices using the edge aggregators. Each IoT device collects data, referred to as ‘messages’, and signs these messages with its private key to authenticate their origin. All these data are then sent to an edge aggregator where some pre-processing is completed using the signatures and actual message to conform with the efficient ECDSA verification method explained in Section 2.3.2. These signed messages are then batched for processing, with each batch being assigned a unique identifier to distinguish it from others. This unique identifier plays a crucial role in the later stages of verification.

For each batch of data, a Merkle tree is constructed. The leaves of this Merkle tree consist of a hash that incorporates the message, its signature, the corresponding public key, and the batch identifier. The construction of the Merkle tree is a critical step as it enables the efficient and secure verification of the data inclusion in the batch. The Merkle tree root and the current batch number are used as the message the aggregator has generated, so it has to sign it. Pre-processing is completed for this message and signature as well, so it can be used as the public input in the proving system (Nova). All other IoT data will be the private inputs. This way, we find a ZK proof that gives no information about the IoT data. The public input, which is the signed Merkle tree root plus the batch number, allows us to prove that a certain IoT data packet was part of the authentication process via signature aggregation.

The size of the aggregated signature, or more exactly, the proof of aggregation, also called the recursive proof, is quite large, so an additional proving is completed to compress the recursive proof. This compressed SNARK proof comes with the Nova prover and is based on Spartan. Essentially, it creates a smaller proof that proves that we have a proof that satisfies the initial statement, in this case, that the aggregation has been completed correctly. The proof is intrinsically linked to the batch identifier, ensuring a clear and verifiable connection between the proof and the specific batch of data.

To enhance the integrity and provide a timestamp for our system, the aggregator records crucial information on a blockchain. This includes the hash of the aggregated signature (compressed cryptographic proof), the Merkle root of the current batch, and the batch identifier. Using a blockchain here is pivotal, as it offers an immutable ledger, ensuring that the data cannot be altered retrospectively once recorded.

To see how the solution would work, imagine a transaction occurs and a buyer purchases specific data from an IoT device. They are provided not just with the message but also its signature, the corresponding public key, the batch identifier, and crucially, a Merkle proof. This Merkle proof enables the buyer to independently verify that the specific message was indeed part of the batch linked to the recorded proof. The buyer can verify this by checking the Merkle proof against the publicly recorded Merkle root and ensuring that the batch identifier aligns with the blockchain record. This verification process confirms that the message was part of the specific batch for which the proof was generated, thus assuring the buyer of the authenticity and integrity of the data. Verification that the proof is correct can also be completed by providing the buyer with the proof (along with necessary verification tools) and them verifying it themself.

Security considerations of this approach are discussed in Section 5.

##### Data Storage

**Figure 3:**
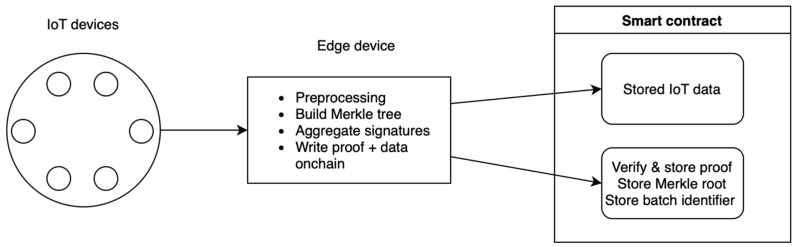
Fully on-chain storage.

The edge aggregating servers also take care of storage (IoT data like sensor readings) by storing them on the aggregator itself, in a cloud database, or completely on-chain. The image below depicts the whole system where the aggregator writes the final proof/aggregate on-chain. The idea here is that once a setup has been created, the aggregator keeps producing new proofs for batches of signatures and then writes each proof and IoT data on-chain. Smart contracts are deployed to store the data and verify the proof. While this approach would be the ideal solution, it is not yet possible as of this paper, with the reason being that the proof and whole data are still too big. Consequently, high gas costs are accrued for writing all of these data. The complexity of creating a verifier smart contract is also an issue. We believe that the exploration of full on-chain storage should still be considered and explored further since rollups on L2 can also be built to store data on cheaper systems than on L1. Perhaps the use of Layer 3 or alternative blockchain storage solutions like EigenLayer, Celestia, Filecoin, and Arweave could also be considered (Figure 3).

**Figure 4:**
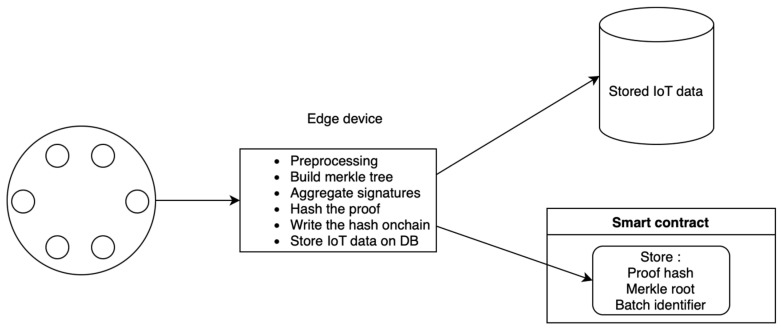
Divided storage (on-chain + off-chain database).

A better approach would be to store all of the IoT data off-chain and only include hashes of the proofs on-chain, working as a timestamp of when the proof was generated and preventing the aggregators from tampering with the proofs. From there, anyone can verify the proofs on their own, which is an acceptable solution for many non-financial privacy applications. The image below depicts such a system (Figure 4).

### 3.2. Proving System Implementation

The implementation and testing of our aggregator started with choosing a proving system that would allow us to skip the trusted setup that comes with some SNARKs. We first utilized the RISC Zero zkVM, a high-performance tool for proving the correct execution of arbitrary code written in Rust. This tool lowers the threshold to achieve a working provable program because we just have to write Rust code and the zkVM proves it. Applications written in Risc0 are structured into two parts: the host code and the guest code. The guest program is the code that we want to run; in our case, it is the ECDSA verification algorithm, and the host code is the code that proves that the guest code was executed correctly. The idea here was to create one proof of verification per signature and lastly create an aggregated proof of proofs with their recursive solution that would compress many Risc0 receipts (proofs) into a single receipt and lastly compress the last receipt via their STARK-to-SNARK circuit that translated a STARK proof into a SNARK proof. Unfortunately, their recursion circuit was not yet available or documented at the time of writing and neither was their compression circuit. Nonetheless, the proving time for one ECDSA signature verification was an eye opener since the proving speed was unsatisfactory for large IoT data authentication. Despite the ease of use of Risc0, the lack of performance led us to Nova, a performant recursive prover.

In Nova, we have to write our own circuits. We re-used the Efficient ECDSA verification circuit written by Personae Labs with some modifications. The circuit was written in Circom, but Nova natively supports the Bellpeperson library for Rust, so we utilized an open source library, Nova-scotia, as the middleware between Nova and Circom. Since we are verifying ECDSA (secp256k1) signatures, we utilized secp/secq curve cycles, which Nova already support. The proving side works over the secq curve and the verifying works over secp, so when compiling with the Circom compiler, we had to specify the prime to be used—prime secq256k1 for the circuit generation.

The outputs are the circuit in R1CS and the witness generator in wasm. We could then write the implementation for Nova in Rust. We read the pre-processed signatures, generated the public parameters using the R1CS file, and lastly, created and verify the proofs.

**Figure 5:**
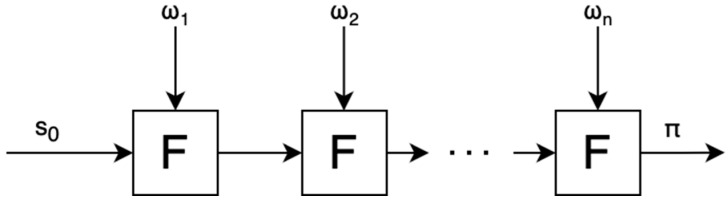
Nova recursive prover.

There are two proving stages: the first is the recursive one that proves the validity of all signatures (signature aggregation), and the second for compressing the recursive proof. The recursive proving takes in the public input s_0_ and the private inputs ω (witnesses), with F being the signature aggregation. The public input is the Edge serve message, signature, and public key, and the private inputs are all of the IoT messages, signatures, and public keys. Both the public and private inputs are pre-processed to conform with the efficient format (Figure 5).

Alongside the actual inputs, the recursive prover also takes the R1CS and witness generator files, as well as the public parameters generated with the R1CS file.

For each aggregation step, the prover will take 10 signatures and then continue to the next step with the next 10 signatures. At the very end, we receive the recursive proof, which is quite large, which is why we further compress this proof with the Spartan prover incorporated with Nova.

### 3.3. On-Chain

#### 3.3.1. Layer 2 Rollups

We have set up a Layer 2 (L2) rollup for our proposed solution, since we believe it presents a superior approach for real-world applications. This preference is due to rollups’ ability to remain centralized and thus gain scalability while still ensuring security by posting proofs and compressed block data on the primary Layer 1 (L1). Initially, we set up our rollup to settle on Ethereum’s test network Holesky to assess usability and functionality.

However, for the actual testing and benchmarking, where costs of interacting with the rollup were recorded, we employed the Optimism Sepolia testnet. This choice was made to ensure more realistic blockchain load conditions, which could not be replicated on a laptop with minimal traffic. The Sepolia testnet provided a more accurate environment for testing, with loads similar to real-world scenarios.

This layered structure, where we can spin up new rollups, facilitates the creation of new modules that can achieve better performance and privacy (with some modifications) while relying on L1 for settlement and security. As one rollup becomes congested, we can simply spin up new rollups, all settling on Ethereum. This approach is also intriguing because we do not silo ourselves from the broader ecosystem, which includes an abundance of open-source developments, liquidity, and user bases. Furthermore, this model provides a standardized means of interoperability with other rollups. For instance, two companies could create their L2s with tailored adjustments and exchange data and resources within the shared ecosystem via bridges or Ethereum itself.

#### 3.3.2. Smart Contract

While the layered blockchain structure allows for increased modularity and scalability by spinning new rollups, additional modularity should be achieved on the smart contract side. We implement this by utilizing the MUD framework [33]. It allows for our proposed system to be scalable, handling potentially vast numbers of IoT devices that are aggregated using multiple edge aggregators.

The MUD framework comprises two primary components: Store and World. Store serves as an alternative to Solidity’s storage engine, offering a data model similar to a relational database or key-value store. This design allows for automatic indexing via event emissions on each storage operation and packs data more compactly than Solidity’s storage engine. Moreover, it enables on-chain reading of external contract storage without depending on existing view functions.

In our implementation, each piece of IoT data (hashes, proofs, and/or the data itself) is stored as a record in a table within Store. Each record is uniquely identified using a combination of a ResourceId (tableId) and a composite key (bytes32[] keyTuple). The ValueSchema of each table defines the data types stored, similar to column types in a database table. The table schema used in our implementation is outlined in Table 1, displaying the schemas for device registration and aggregator data entry. As the network of IoT devices grows, requiring more edge aggregators, our system can expand by creating multiple namespaces with these tables. Access control for each namespace ensures that only specific edge aggregators can write batches.

Store also automatically generates a library for each table, providing getter and setter functions. Furthermore, Store allows for runtime schema definition, enabling the registration of new tables with new schemas after deployment. This capability is crucial for advanced applications such as the World protocol, which we utilize in our IoT data authentication framework.

The World component of MUD provides the logic and access control layer on top of Store’s storage capabilities. It acts as a central entry point for calls, performing access control checks and routing authorized requests to the appropriate System. Systems are stateless contracts interacting with data in Store. They can read from all tables and modify data in accessible tables, typically within their namespace, which can be understood as containers for tables and systems. Even access control is based on namespaces.

Our experimentation involved several steps. Initially, we defined the table schemas. This was followed by the implementation of system contracts, utilizing the libraries generated for each table. We crafted a single system contract containing functions to modify the data in both tables. Subsequently, we deployed the smart contracts using the MUD framework. After deployment, our focus shifted to logging the gas costs associated with device registration and the entry of edge aggregator data for specific batches. The entire process was straightforward, effectively highlighting the user-friendly nature of the MUD framework.

**Figure 6:**
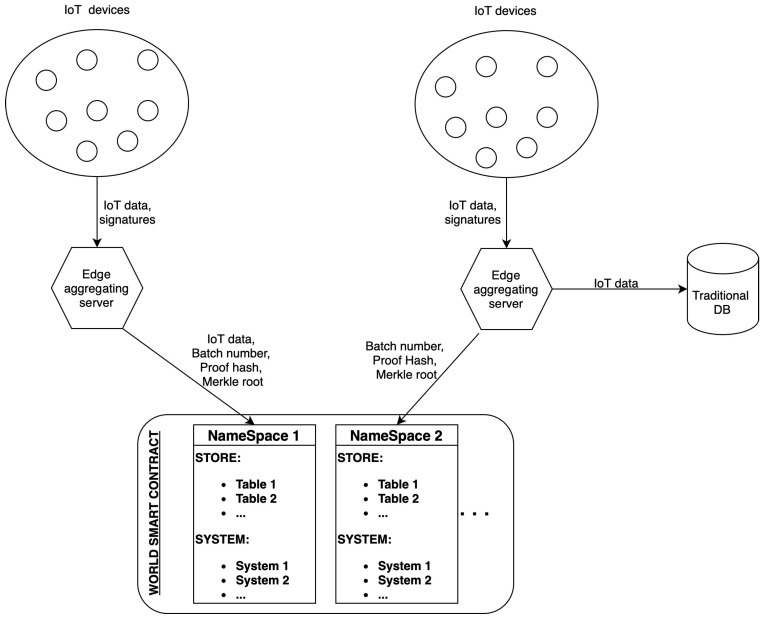
Overview of the whole solution (smart contract + off-chain aggregation).

Figure 6 depicts the complete solution architecture. The process begins with multiple IoT devices collecting data and generating corresponding digital signatures. These data, along with the signatures, are then transmitted to the edge aggregating servers.

On the left, we have illustrated how a fully on-chain storage approach would look like, but it is important to note that we have not tested this approach. We have instead defined alternative research approaches in the Conclusion section (Section 6) that focus on a fully on-chain approach. The edge aggregator receives data and signatures from its connected IoT devices. It performs signature aggregation to create a compact batch that includes aggregated IoT data, the batch number, proof hash, and Merkle root. This batch is then written to NameSpace 1 on the blockchain for immutable storage, ensuring integrity and verifiability of the data. On the right, another edge aggregator similarly receives data and signatures but opts for a different data handling approach. After aggregation, the IoT data are forwarded to a traditional database for storage, while only the batch number, proof hash, and Merkle root are written to NameSpace 2 on the blockchain. This method segregates the detailed IoT data from the blockchain, leveraging traditional databases for storage, and uses the blockchain primarily for verification purposes.

Below the aggregators, the diagram shows the World Contract, which acts as the backbone of the system’s on-chain component. It houses NameSpace 1 and NameSpace 2, each containing tables for storing data and systems for executing logic.

## 4. Results

Our solution is structured into two main components, resulting in our findings being split into two distinct sections: off-chain and on-chain. Our experimental setup for the off-chain (edge aggregating server) component was carried out using a MacBook M1 Pro with 16 GB of memory, which facilitated the creation of signatures, their verification, and the generation of proofs. Our framework is designed to be compatible with any IoT device capable of securely creating signatures. This includes devices that can safely store and use private keys. For the on-chain component, we utilized the public Optimism Sepolia testnet, rather than our own rollup, to simulate more realistic blockchain load conditions and provide a more accurate assessment of on-chain costs.

The off-chain portion elaborates on the signature aggregator’s proving times and proof sizes. This aggregator processes IoT messages and signatures, compiling them into batches, generating succinct proof of verification/aggregation. In this context, we have explored two proving systems, both employing a transparent approach that eliminates the need for a trusted setup. We present a comparison of the outcomes achieved by these two systems. On the other hand, the on-chain section delves into the storage costs and the registration process for devices, encompassing both edge aggregators and IoT devices. Our tests on the on-chain component focused solely on recording the essential data needed to authenticate an entire batch after its proof. While direct on-chain storage of all IoT data has not been evaluated, the costs can be inferred from the expenses incurred in storing only the necessary batch data.

### 4.1. Proving Times and Proof Sizes

**Figure 7:**
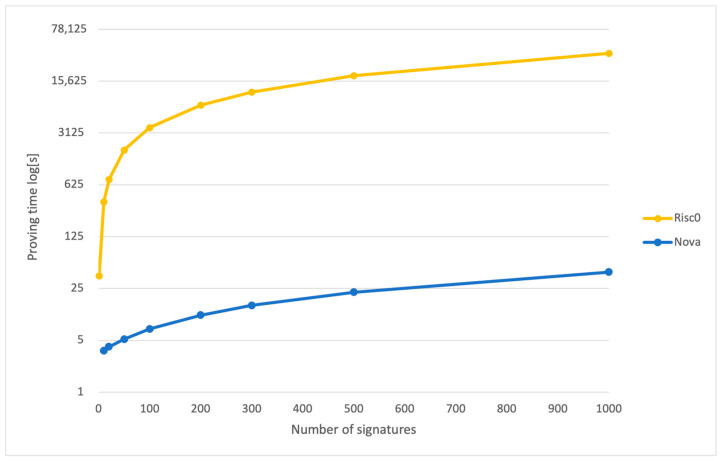
Proving time for Nova (recursive + compressed) and Risc0.

Figure 7 illustrates the comparative proving times for the Nova and Risc0 systems as the number of signatures required for aggregation increases. For Nova, the proving times represent the total of two components: the time required to generate the recursive SNARK and the time to produce a smaller, compressed SNARK derived from the recursive one.

**Figure 8:**
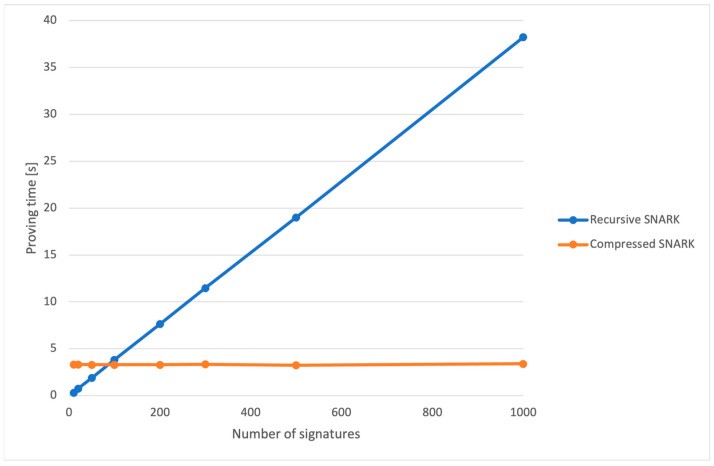
Nova proving time for the recursive and the compressed SNARK.

Figure 8 presents the proving times for the recursive and compressed SNARKs in the Nova prover. We can see that the time for the compressed SNARK remains constant, while the recursive one rises linearly with the increase in the number of signatures. This is because the compressed one only proves the correctness of the recursive SNARK regardless of the number of signatures, while the recursive one has to aggregate more signatures.

The recursive proof in Nova is approximately 8.7 MB, while the compressed proof is just 29, in contrast to the 1.3 MB size of the Risc0 proof. However, it is important to note that the Risc0 proof is not compressed, so for our case, aggregating multiple proofs and then compressing the last proof would be necessary.

**Figure 9:**
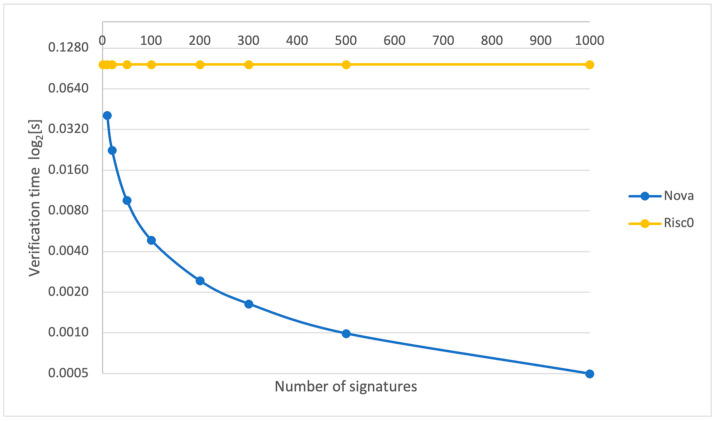
Verification time per signature for Nova (recursive + compressed) and Risc0.

While proving time is important on the edge aggregating server side, verification times are crucial for clients who need to verify that the aggregation and, thus, data authentication have been correctly performed. Figure 9 depicts the verification time per signature for both the Risc0 and the Nova provers. We have combined the verification times for the Nova prover for the recursive and compressed SNARKs. It can be observed that the verification times per signature remain constant for the Risc0 prover, which is expected as a proof is created for each signature verification.

**Figure 10:**
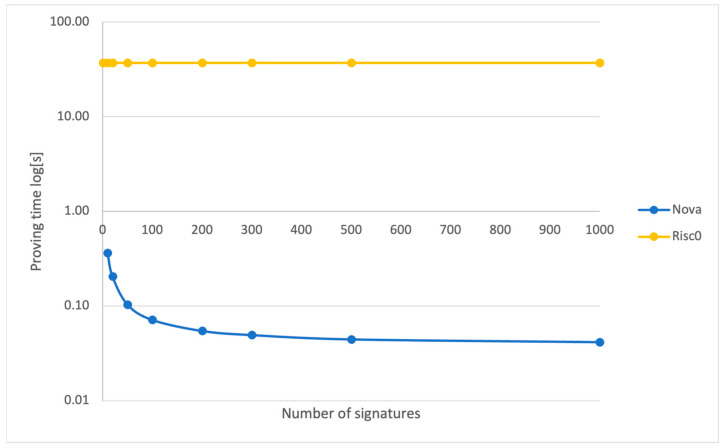
Proving time per signature for Nova and Risc0.

Analyzing the proving times per individual signature is equally important. That is why Figure 10 illustrates the proving times for both the Nova and Risc0 proving systems on a per-signature basis. It shows that the proving times for Nova decrease as the number of signatures in a batch increases, indicating improved efficiency at scale. In contrast, the proving times for Risc0 remain constant, which is consistent with its overall proving time trend.

### 4.2. On-Chain Storing Costs

For our on-chain test, we assessed the costs associated with registration and the cost of writing authenticating data for each batch, which includes the batch number, aggregating proof hash, and the Merkle root. We present the costs for Layer 1 (L1) and Layer 2 (L2) networks, demonstrating how using L2 can significantly reduce costs.

Two factors contribute to gas consumption within L2 networks, specifically in our case, with a rollup based on the Optimism stack. The first is the portion of gas used for writing our transaction to L1, and the second is the gas used for executing the transaction on L1. The gas prices for these two components differ.

Equations (1) and (2) depict how the transaction fee is calculated based on the gas used for the transaction and the current gas price for both networks. This calculation includes the base gas price, the priority gas price, and a scalar representing a dynamic overhead cost, which, at the time of writing, was set to 0.684. To determine the actual transaction fee, both fees from L1 and L2 must be summed.

(1) $$transaction\;fee\;on\;L2=gas\;used\cdot \left(base\;price+priority\;price\right)$$

(2) $$transaction\;fee\;on\;L1=gas\;used\cdot \left(base\;price\right)\cdot scalar$$

The prices for the gas units and ETH utilized in the calculations are detailed in Table 2, which reflects the average market rates for the Ethereum and Optimism networks at the time of writing, with 1 ETH being equivalent to 10^9^ gwei units.

Table 3 and Table 4 detail the costs of a single registration and data writing. The data are presented in terms of gwei units and US dollars for two distinct scenarios: one using the Ethereum mainnet and the other employing a rollup based on Optimism.

**Figure 11:**
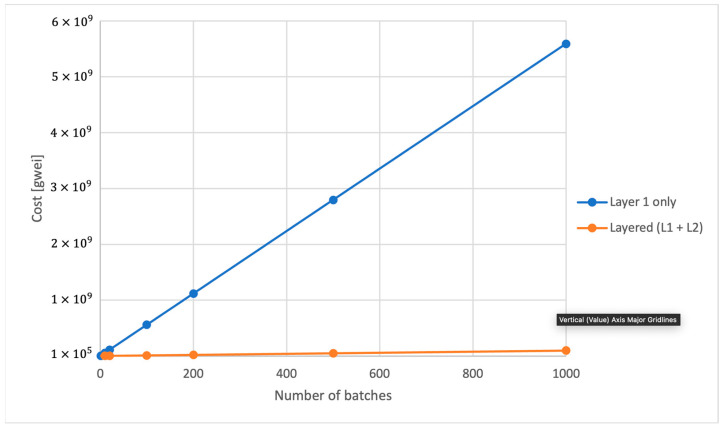
Cost comparison between using Layer 1 only and Layered (L1 + L2) for writing batch authenticating data.

Figure 11 depicts the difference in accumulated costs for the scenario where we write the authenticating data for a batch of signatures. It shows how the costs of utilizing L1 instead of L2 would rise dramatically as the amount of data written for batches rises or accumulates over time.

## 5. Discussion

IoT data authentication, traditionally centralized, has been revolutionized using blockchain technology, offering immutability and enhanced security. However, while secure, direct interaction with public ledgers is not economically scalable due to transaction costs. Permissioned blockchains improve scalability and privacy, but at the cost of true decentralization, reintroducing a degree of trust and thus lower security.

Our approach tackles this problem by preserving high security while still making the system highly scalable. The solution consists of an off-chain edge aggregating server that manages IoT devices and handles authentication via signature aggregation, coupled with a layered blockchain structure. The first layer includes the Ethereum network, providing high decentralization and security, while the second layer comprises rollups with higher performance and cheaper fees. These rollups are used for device registration—encompassing IoT devices and Edge aggregation servers—and for storing authenticating data for each batch of signatures the edge aggregator processes. These data include the proof hash produced by the proving system inside the edge aggregator, the Merkle tree root, and the current batch number.

### 5.1. Off-Chain Results

We utilized the Nova and Risc0 provers, with Nova being used to aggregate batches of signatures and produce a proof at the end of aggregation and Risc0 to create a proof of signature verification for one signature only. Figure 7 provides a logarithmic comparison of the proving times for Nova and Risc0 as the number of signatures increases. Utilizing a log scale is crucial to effectively visualize Risc0’s performance in conjunction with that of Nova, given that Risc0’s proving times escalate much faster with an increasing number of signatures. For example, when the count of signatures rises from 10 to 20, Risc0’s proving time nearly doubles from 369.7 s to 739.4 s, while Nova’s proving time grows from 3.62 s to only 5.187 s.

The substantial performance gap arises because generating a proof for each signature verification is computationally intensive. Risc0 operates as a non-recursive prover, creating an individual proof for each signature verification. In contrast, Nova functions as a high-performance recursive prover, verifying batches of signatures and producing a proof only after all verifications are complete. This difference in approach underscores the scalability and efficiency of Nova, particularly in scenarios with a large number of signatures.

This gap becomes more pronounced when comparing both systems for proving times and verifying times per signature (Figure 10 and Figure 11), where the Nova prover demonstrates increased efficiency with more signatures, unlike the constant times of the Risc0 prover.

However, the Nova prover’s efficiency comes with a complexity: it has two contributors to proving time. The first is the time it takes to create a recursive proof, and the second is the time required to compress it. These two contributions are there because the proofs created from recursion were too large, so another step of proving the recursive proof was used, creating a final compressed proof which is much smaller in size. For instance, the recursive proof in Nova is approximately 8.7 Mb, while the compressed proof is just 29 Kb, in contrast to the 1.3 Mb size of the Risc0 proof. In Figure 8, we noticed that the proving time for the recursive side grew linearly as the number of signatures being aggregated rose, while the time for compressing the proof stays the same. This makes sense, since the compressing part always takes in one proof regardless of the number of signatures.

It is important to note that the proofs generated with Risc0 could be combined into a single succinct proof. However, we chose not to pursue this due to incomplete Risc0 documentation on this subject and the lengthy proving time for one signature verification. The Nova prover was thus chosen as it provided excellent performance, with proving and verification times per signature decreasing as the number of signatures in a batch being processed grew.

### 5.2. On-Chain Results

For the on-chain results, we measured the costs associated with registering devices and writing the authenticating data from the edge aggregator for one batch, irrespective of the number of signatures in a batch, as the proof hash and Merkle root sizes remain constant. The results demonstrate the cost advantage of using Layer 2 rollups over Ethereum Layer 1. Specifically, the costs of using Layer 1 only are almost 48 times larger than using Layer 2 for device registration and 57 times larger for authenticating data writing. Thus, employing a Layer 2 rollup significantly reduces costs compared to using the Ethereum mainnet. This cost reduction is in addition to the decreased costs for IoT devices, which, in our approach, do not need to interact with a ledger. Yet, the proving system and the Merkle tree root guarantees that the data are authentic, originating from registered IoT devices, and have not been tampered with.

### 5.3. Security Discussion

With our approach leveraging cryptographic proofs (SNARKs) and Merkle trees, we have established a foundation for secure and privacy-focused IoT data authentication and storage. This method inherently enhances security: any tampering with the input data or the prover would result in the generation of invalid proofs, thereby signaling potential security breaches and ensuring that only valid data are processed and recorded.

Nevertheless, for a system to be production-ready, other security considerations must be taken into account. While our research assumes the security of IoT devices and edge servers at both the hardware and software levels, the practical implementation of such systems in real-world scenarios may present additional challenges. Ensuring the physical and digital integrity of IoT devices is crucial, as they are often deployed in uncontrolled environments and could be prone to tampering or unauthorized access. Similarly, edge servers, while assumed to be secure in our framework, would require rigorous security protocols to prevent data breaches, unauthorized access, and ensure data privacy.

Another critical aspect is the secure communication between IoT devices and edge aggregating servers. It is essential to safeguard data transmission to prevent interception, eavesdropping, or manipulation. Implementing robust encryption protocols for data in transit, such as TLS/SSL, along with mutual authentication, integrity checks, and access controls, can provide the necessary multi-layered security.

Further research should explore these areas in depth, focusing on robust methods to secure IoT devices and edge servers against a wide range of threats. One potential avenue is the integration of automated security checks that could continuously monitor for signs of tampering or unauthorized access, both in IoT devices and at the server level. Such mechanisms would be particularly effective given the security guarantees our approach allows. The use of SNARKs and Merkle trees not only enhances data integrity but also offers a solid foundation for developing additional security features.

Furthermore, while the approach is discussed in Section 3.1.2 using Merkle trees enhances the verifiability of the data, there is still an element of trust in the server. The server’s role in constructing the Merkle tree and generating the public input for the prover must also come with the guarantee that the private inputs going into the prover are the same data being used to build the Merkle tree. As of this paper, this issue has not yet been tackled, but some approaches we foresee could be as follows:

1. Modifying the Prover.
  - Modify the prover to take public keys as inputs and signatures and messages as private inputs. This could provide a more direct link between the data sources (IoT devices) and the proof.
  - Modify the prover to construct the Merkle tree as part of the proof generation process. This would tightly couple the data verification with the proof itself, providing strong assurance that the same data are used in both the Merkle tree and the proof, without the need to disclose any data for each batch.
2. Proving that the process of building a Merkle tree used the same data as the process of aggregating signatures. This could be completed by utilizing yet another proving system or for example, Intel’s trusted execution environment (SGX).

## 6. Conclusions

In contrast to existing IoT data authentication methods described in Section 2.1, our solution offers a unique blend of efficiency, scalability, and privacy. Unlike approaches that primarily rely on post-storage data integrity checks or over-dependence on edge servers, our framework integrates zk-SNARKs and Merkle trees to ensure data privacy with verifiability. This integration not only maintains privacy but also enables the verification of data origin and integrity. Furthermore, our framework’s design for scalability, utilizing edge aggregating servers, addresses the common issue of computational overhead in resource-constrained IoT devices, a significant limitation in many existing techniques. By recording key information such as the Merkle root and proof hash on the blockchain, our approach also ensures immutable and transparent record keeping, enhancing trust and authenticity far beyond what is typically achieved in the current literature. Additionally, our approach to authentication is more performant than similar approaches utilizing ECDSA batch verification.

In essence, the solution shifts the bottleneck to an off-chain system, moving the latency associated with IoT data authentication (signature verification) to this off-chain system. Rollups allow for almost immediate soft transaction confirmation, with the proving time of edge aggregators becoming the main contributor to latency.

Using recursion in our chosen proving system is crucial, as it improves proving time performance. Additionally, proof compression is vital, and without it, the proofs are too large for practical use.

While current results in proving times and proof sizes show that a combination of recursive proving and compression is necessary, we anticipate further advancements and refinements in proof generation. These improvements will likely reduce proof sizes and times even more, allowing us to store the compressed proofs directly on-chain more easily. This would make using smart contracts to verify the proofs easier, enhancing immediacy and integration within the blockchain ecosystem and transitioning our solution from a Layer 2.5 to a Layer 3 system.

Future work could be considered in implementing aspects described in the Security discussion. Another avenue to explore would be other L2 solutions as they mature. While rollups store all transaction data on L1, other L2 solutions like plasma, validiums, and volitions offer different data storage mechanisms, potentially reducing costs even further. Plasma, for example, keeps most data and computation off-chain, except for critical components like deposits, withdrawals, and Merkle roots [34]. Validiums are similar to ZK-rollups but store data off-chain, relying on Data Availability Committees for data storage [22]. Volitions, pioneered by StarkWare, allow applications to switch between ZK-rollup and validium modes, offering flexibility regarding on-chain and off-chain data storage [35]. These solutions present interesting avenues for blockchain-only solutions in IoT data authentication, providing significant cost reductions while maintaining security and integrity.

## Figures and Tables

**Table 1:** Schema for our data tables.

Device Registration	Aggregator Data Entry (Batch Info)
keySchema: { owner: “address”, }, valueSchema: { isIotDevice: “bool”, },	keySchema: { owner: “address”, }, valueSchema: { batchNumber: “uint32”, proofHash: “string”, merkleRoot: “string”, },

**Table 2:** Prices used in cost calculations.

Layer 1		Layer 2		
Base Price [gwei/gas]	Priority Price [gwei/gas]	Base Price [gwei/gas]	Priority Price [gwei/gas]	ETH Price [USD/ETH]
28	0.1	0.00345	0.02	2150

**Table 3:** Gas usage and cost for device registration in the case of using L1 or L2.

	Layer 1 Only	Layered (L1 + L2)	
	Gas Used	Gas Used L1	Gas Used L2
Registration	74,653	2188	74,653
Cost in gwei and USD			
Cost [gwei]	2,097,749.3	41,904.576	1750.612
Cost [USD]	4.51	0.090	0.003
Cost total [USD]	4.51	0.093	

**Table 4:** Gas usage and cost for batch data writing in the case of using L1 or L2.

	Layer 1 Only	Layered (L1 + L2)	
	Gas Used	Gas Used L1	Gas Used L2
Writing data	198,979	4820	198,979
Cost in gwei and USD			
Cost [gwei]	5,591,309.9	92,312.64	4666.057
Cost [USD]	12.02	0.198	0.010
Cost total [USD]	12.02	0.208	

## Data Availability

The original contributions presented in the study are included in the article, further inquiries can be directed to the corresponding authors.
